# Natural History and Reintervention After PCNL for Isolated Lower-Pole Stones with Residual Fragments > 4 mm: A Retrospective Cohort Study

**DOI:** 10.3390/jcm15187060

**Published:** 2026-09-11

**Authors:** Ekrem Akdeniz, Mücahid Uğur, Ömer Dağlar, Reha Ordulu, Emrah Küçük, Muhammed Esad Kayhan, Mesut Şengül, Muhammed Bahattin Ulu, Mahmut Ulubay, Mustafa Kemal Atilla

**Affiliations:** 1 Department of Urology, Faculty of Medicine, Samsun University, 55080 Samsun, Türkiye; 2 Department of Urology, Samsun City Hospital, Samsun University, 55080 Samsun, Türkiye; 3 Department of Urology, Gazi State Hospital, 55070 Samsun, Türkiye

**Keywords:** lower-pole stone, percutaneous nephrolithotomy, residual stone, urolithiasis

## Abstract

**Background:** This study aimed to describe the long-term clinical trajectory, stone burden changes, and secondary endourological reintervention outcomes in patients presenting with residual stone fragments (RSFs) > 4.0 mm confined to the lower pole following percutaneous nephrolithotomy (PCNL). **Methods:** We retrospectively reviewed a single-center cohort of 29 consecutive patients who underwent index-PCNL for isolated lower pole calculi and presented with RSFs > 4.0 mm on routine non-contrast computed tomography (NCCT) at 3 months postoperatively (defined as study baseline and time zero), followed for a minimum of 12 months. Demographic data, baseline and terminal stone burden, stone-related clinical events, procedural complications, and secondary endourological interventions were analyzed. **Results:** Twenty-nine patients (20 males, 9 females; mean age: 50.1 ± 12.0 years) were analyzed. Preoperative median two-dimensional (2D) stone burden was 332.0 mm^2^ (IQR: 282.0–386.0 mm^2^). At the 3-month baseline NCCT, median residual 2D stone burden was 24.0 mm^2^ (IQR: 13.0–33.0 mm^2^). Over a median follow-up of 36.0 months (IQR: 18.0–48.0 months), four patients (13.8%, 95% CI: 3.9–31.7%) achieved spontaneous stone passage, 15 patients (51.7%, 95% CI: 32.5–70.6%) required secondary reintervention due to interval enlargement or symptoms, and 10 patients (34.5%, 95% CI: 17.9–54.3%) remained on active surveillance. Across the cohort, median stone burden on the last positive scan prior to clinical events or the final surveillance visit increased to 57.0 mm^2^ (IQR: 38.0–104.0 mm^2^). **Conclusions:** In this selected single-center cohort of 29 patients with lower-pole residual fragments > 4.0 mm at 3-month NCCT after PCNL, 15 patients underwent secondary intervention and four experienced documented spontaneous passage during variable follow-up. Because interval monitoring and intervention criteria were individualized, these findings are descriptive and do not establish the optimal timing of treatment. Larger prospective studies with standardized imaging protocols are required to better define management strategies.

## 1. Introduction

Lower pole calculi account for 25.0–50.0% of all renal stones, exhibit lower rates of spontaneous clearance, and frequently necessitate active intervention [1,2]. Although multiple therapeutic modalities are available, pelvicalyceal anatomical factors—such as infundibular length, infundibular width, and the infundibulopelvic angle—in conjunction with patient- and stone-specific characteristics, make the management of lower-pole stones particularly challenging [1,3]. The European Association of Urology (EAU) guidelines maintain specific recommendations for lower pole calculi, endorsing percutaneous nephrolithotomy (PCNL) as the primary modality for stones measuring >20.0 mm, while for stones 10.0–20.0 mm in size, shock wave lithotripsy (SWL), retrograde intrarenal surgery (RIRS), or PCNL is recommended based on individual stone characteristics and calyceal spatial anatomy [4]. For large lower-pole stones, PCNL remains highly effective, with randomized controlled trials reporting success rates ranging from 76.0% to 98.3% [5].

Residual stone fragments (RSFs) following endourological procedures or SWL present a substantial therapeutic dilemma, which is further accentuated in the lower pole due to dependent gravity and unfavorable drainage dynamics [2,6]. Residual fragments ≤4.0 mm have traditionally been categorized as clinically insignificant; however, accumulating contemporary evidence increasingly questions this distinction, demonstrating that even small fragments can undergo interval enlargement, cause recurrent symptoms, or necessitate delayed reintervention [6,7,8]. Conversely, fragments > 4.0 mm are widely recognized to carry an even higher risk of progression and clinical complications [6,7]. Current EAU guidelines provide a weak recommendation regarding the active removal of RSFs > 4.0 mm following endourological interventions, while acknowledging conservative surveillance as an acceptable alternative in selected asymptomatic patients [4].

Although the previous literature has documented low spontaneous passage and high intervention rates for residual calculi in general, real-world longitudinal evidence restricted specifically to isolated lower pole RSFs > 4.0 mm following PCNL remains scarce. Because dependent lower pole calyceal anatomy naturally hinders clearance and fosters crystal aggregation, defining the long-term clinical trajectory of these calculi under routine clinical surveillance provides valuable descriptive evidence within the framework of existing guideline recommendations. Therefore, the aim of this retrospective study was to describe the longitudinal clinical course, quantitative stone burden changes, and secondary endourological intervention requirements in a single-center cohort of patients presenting with lower pole RSFs > 4.0 mm following PCNL.

## 2. Materials and Methods

### 2.1. Study Design

This retrospective observational study involved patients who underwent PCNL due to lower-pole stones at the Samsun University Samsun City Hospital Urology Clinic, Türkiye. Ethical approval was granted by the Samsun University Non-Interventional Clinical Research Ethics Committee (Decision No: GOKAEK 2025/20/7, Date: 15 October 2025) prior to data extraction, in accordance with the Declaration of Helsinki. The recruitment window for index-PCNL procedures spanned from January 2015 through September 2024. Because a minimum follow-up duration of 12 months was required, postoperative monitoring continued beyond the interventional window through 30 September 2025. Follow-up records were systematically retrieved and verified between 16 October and 30 November 2025, and the database was formally locked on 31 December 2025. All consecutive PCNL cases performed during the study period were retrospectively screened. The final cohort comprised patients with isolated lower-pole stones who presented with RSFs measuring >4 mm at the 3-month postoperative evaluation.

Inclusion criteria:

- Patients aged over 18;
- Patients undergoing PCNL for the first time due to lower-pole stones;
- Patients followed up for longer than 12 months.

Exclusion criteria:

- Undergoing PCNL due to pelvic, middle, or upper pole stones;
- Patients with residual stones falling into the lower pole during the application of PCNL other than to the lower pole;
- Patients with previous histories of PCNL or open renal surgery to the same kidney;
- Patients with follow-up times shorter than 12 months;
- Patients with skeletal deformity;
- Patients with a pelvic kidney or rotation anomaly.

**Figure 1:**
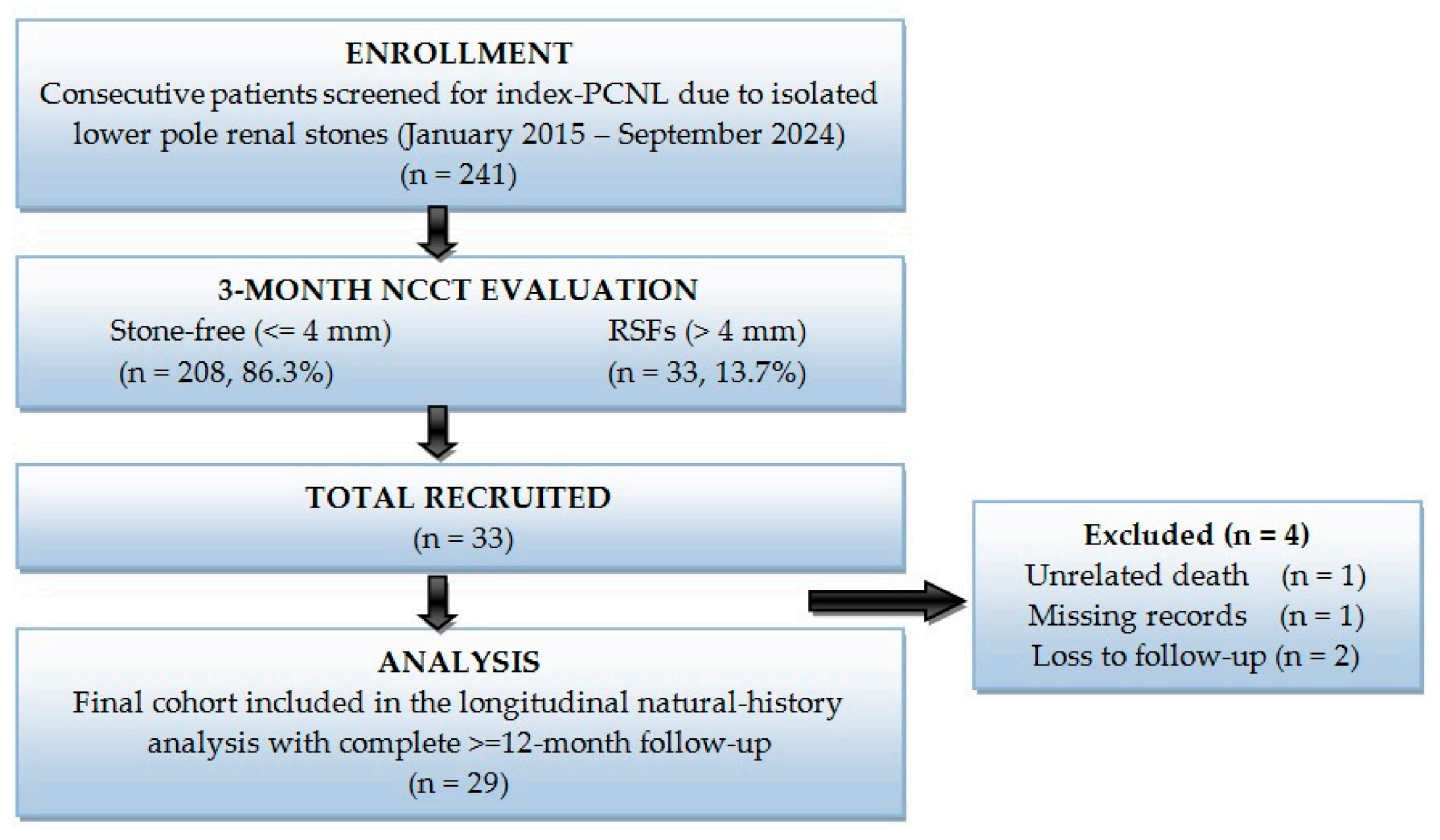
STROBE participant flow diagram (PCNL, percutaneous nephrolithotomy; NCCT, non-contrast computed tomography; RSFs, residual stone fragments).

A total of 241 consecutive patients (241 renal units; one unit per patient) who underwent index-PCNL for isolated lower pole renal stones during the study period were initially evaluated. At the 3-month postoperative non-contrast computed tomography (NCCT) assessment, a stone-free status was achieved in 86.3% of cases (*n* = 208), whereas RSFs (>4 mm) were detected in 33 patients (13.7%). Of these 33 patients, 4 were excluded from the longitudinal analysis due to death unrelated to surgery during follow-up (*n* = 1), missing baseline clinical records (*n* = 1), and loss to follow-up/non-attendance at subsequent visits (*n* = 2). Consequently, the final analysis included 29 patients with complete clinical and radiological follow-up datasets (Figure 1). Patients’ sociodemographic characteristics, American Society of Anesthesiologists (ASA) scores, clinical and morphological stone characteristics, operative times, and lengths of hospitalization were recorded. Peri- and postoperative complications, residual fragment dimensions, stone progression during follow-up, and additional intervention requirements were also entered retrospectively into the database.

### 2.2. Preoperative Evaluation

As part of standard clinical care, written informed consent detailing the surgical procedure and potential perioperative complications was obtained from all patients prior to surgery, and all underwent routine preoperative biochemical, hematological, and serological evaluations along with complete urinalysis. All patients were screened with preoperative urine cultures; those with positive cultures were treated with pathogen-directed antimicrobial therapy, and surgery was scheduled only after confirming sterile urine on repeat culture. Patients with confirmed sterile urine received intravenous second-generation cephalosporin prophylaxis at anesthesia induction in accordance with antimicrobial guidelines. NCCT (1.0–1.25 mm slice thickness with multiplanar coronal and sagittal reconstructions) was performed preoperatively and routinely at the 3-month postoperative evaluation, which served as the baseline for RSF analysis. Study eligibility was defined strictly by the presence of a dominant residual fragment measuring > 4.0 mm in its longest axis on the 3-month NCCT. Patients presenting solely with clinically insignificant fragments (≤4.0 mm) were excluded, even if multiple fragments were present. All measurements were conducted on bone window settings by an experienced uroradiologist. For each fragment, the longest diameter (L, mm) and the greatest perpendicular diameter (W, mm) in the same cross-sectional reconstruction plane were measured. The two-dimensional (2D) stone burden was determined as length multiplied by width (L × W, mm^2^). In eligible patients with multiple residual fragments, the cumulative 2D stone burden was calculated as the sum of the products of all individual fragments (∑ [L × W]). During longitudinal follow-up, new de novo stone formation was documented and analyzed separately from the linear and two-dimensional progression of baseline fragments. All stone parameters are consistently reported to one decimal place. Stone density was measured in Hounsfield units (HU). These values were calculated using the average attenuation measurements obtained from the central and peripheral regions in the section in which the stone was best visualized at NCCT.

### 2.3. Surgical Technique

All index-PCNL procedures were performed under general anesthesia with the patient in the prone position by two experienced academic endourologists. Following the retrograde placement of a 5 Fr ureteral catheter (Geotek Medikal, Ankara, Türkiye), a single lower-pole calyceal access was established under C-arm fluoroscopic guidance in all 29 patients (100.0%) using an 18-gauge access needle (Boston Scientific Corp., Natick, MA, USA). After obtaining clear urinary return, a guidewire (Sensor™ Guide Wire, Boston Scientific Corp., Marlborough, MA, USA) was advanced into the pelvicalyceal system. Serial fascial tract dilation was performed over the guidewire using Amplatz dilators (Boston Scientific Corp., Marlborough, MA, USA) to 24 Fr (*n* = 6, 20.7%), 26 Fr (*n* = 9, 31.0%), 28 Fr (*n* = 10, 34.5%), or 30 Fr (*n* = 4, 13.8%). Rigid nephroscopy (Karl Storz, Tuttlingen, Germany) combined with pneumatic lithotripsy was uniformly utilized for stone fragmentation and subsequent forceps extraction; flexible nephroscopy was not routinely deployed during the index procedures. Intraoperative stone clearance was assessed through direct rigid nephroscopic visualization and multi-angle fluoroscopic screening. At the conclusion of the procedure, a nephrostomy catheter was placed as an exit strategy, clamped to evaluate ureteral patency and peritubal leakage prior to removal, and removed prior to discharge on postoperative day 2 or 3.

### 2.4. Assessment of Complications

Adverse clinical events were systematically categorized into three distinct chronological phases: (a) perioperative complications of the index-PCNL procedure (≤30 days postoperatively); (b) stone-related clinical events arising from RSFs during longitudinal surveillance (e.g., acute renal colic, fragment migration, hematuria); and (c) complications associated with secondary endourological re-interventions. Procedural complications were evaluated using the modified Clavien–Dindo classification, focusing on clinically significant events (≥Grade III). Under this grading system, interventional procedures under anesthesia were classified as Grade III (local anesthesia IIIa, general anesthesia IIIb), life-threatening single- or multi-organ failure requiring intensive care unit admission as Grade IV (single organ IVa, multiorgan IVb), and mortality as Grade V [9].

### 2.5. Postoperative Evaluation

All patients’ complete blood counts and renal function tests were evaluated in the early postoperative period. On postoperative day 1, plain radiography of the KUB was performed to verify catheter positioning and inspect gross radiopaque clearance. On postoperative day 1 or 2, the nephrostomy tube was temporarily clamped for 4–6 h to verify normal antegrade ureteral drainage; in the absence of flank pain, fever, or peritubal urinary leakage, the tube was removed prior to discharge on postoperative day 2 or 3.

At 3 months postoperatively, all patients underwent NCCT which established study eligibility and provided the definitive baseline 2D stone burden measurement (study time zero). At this baseline assessment, patients presenting with lower pole RSFs > 4.0 mm were counseled regarding active secondary intervention versus active surveillance within a shared decision-making framework. During longitudinal follow-up, interval surveillance was conducted at 3- to 6-month intervals using plain KUB and renal ultrasonography (USG) to minimize cumulative ionizing radiation in accordance with ALARA principles. However, the definitive final clinical endpoint and terminal stone burden for all 29 patients (100.0%) were established by non-contrast CT: confirming complete clearance following reported spontaneous passage (*n* = 4), for pre-operative reintervention planning (*n* = 15), and at the terminal surveillance visit for conservatively followed patients (*n* = 10).

Following the baseline evaluation, patients entered an individualized surveillance protocol. Routine interval monitoring was conducted at 3- or 6-month intervals using plain KUB and renal ultrasonography (USG) to minimize cumulative ionizing radiation in accordance with ALARA principles. However, the definitive final clinical endpoint and terminal stone burden for all 29 patients (100.0%) were established by NCCT. Specifically, NCCT was mandated to confirm complete clearance following reported spontaneous passage (*n* = 4), for pre-operative surgical planning and stone burden measurement in patients requiring secondary intervention (*n* = 15), and for all patients in the active surveillance group (*n* = 10), in whom the entire longitudinal follow-up was conducted exclusively using serial NCCT. Consequently, all longitudinal 2D stone burden comparisons represent uniform, paired CT-to-CT evaluations. Radiological progression was defined a priori as a ≥20% increase in 2D stone burden relative to the 3-month baseline NCCT, whereas fragment stability was defined as a <20% change in 2D stone burden without new stone formation. Absolute change (Δ, mm^2^) was calculated for all patients. Detailed patient-level longitudinal data are provided in Supplementary Table S1. For patients achieving spontaneous clearance, the definitive terminal stone burden was defined as 0.0 mm^2^, and the last positive scan before passage was documented separately as the pre-event stone burden. Secondary endourological intervention was indicated under emergency conditions for acute intractable renal colic, obstruction, or urosepsis (*n* = 2, 13.3%). For elective cases (*n* = 13, 86.7%), intervention was triggered either by documented radiological stone progression (defined as a >20.0% increase in 2D stone burden or fragment expansion ≥10.0 mm; *n* = 8) or by recurrent/persistent stone-related flank pain or hematuria (*n* = 5). The choice of secondary procedure was determined by interval stone burden and calyceal anatomy: RIRS (*n* = 11) was the preferred minimally invasive approach for moderate stone burdens, whereas repeat PCNL (*n* = 4) was reserved for large aggregate stone burden (≥20.0 mm) or unfavorable infundibulopelvic anatomy that precluded retrograde access. SWL was not offered due to unfavorable lower pole location and high mean stone radiodensity (1105.7 HU).

### 2.6. Medical Follow-Up and Stone Prevention

During longitudinal follow-up, all patients received standardized conservative stone-prevention counseling emphasizing adequate fluid intake (>2.5 L/day) and dietary moderation. Serial urinalyses and urine cultures were monitored at interval visits, with positive cultures treated using targeted culture-specific antibiotics. Specific pharmacological metaphylaxis (e.g., potassium citrate) was not systematically administered owing to the lack of routine 24-h metabolic profiling.

### 2.7. Statistical Analysis

Statistical analyses were conducted using IBM SPSS Statistics for Windows, Version 21.0 (IBM Corp., Armonk, NY, USA), and R software (Version 4.3.2; R Foundation for Statistical Computing, Vienna, Austria). Missing data were handled using a complete-case approach; patients with missing baseline documentation (*n* = 1) or those lost to follow-up (*n* = 2) were excluded prior to analysis, resulting in a final complete dataset of 29 patients (Figure 1). As this study utilized a descriptive observational cohort design and subgroups represented post-hoc longitudinal outcome trajectories rather than prospective comparative treatment arms, formal between-group inferential hypothesis testing was deliberately not performed to avoid outcome-allocation bias. Data across all categories are reported purely as descriptive summary statistics. The distributional characteristics of continuous variables were evaluated to determine appropriate summary statistics. Variables demonstrating normal distribution are presented as mean standard deviation (SD), whereas skewed parameters (specifically 2D stone burden, absolute stone change, and duration of follow-up) are reported as median and interquartile range (IQR: 25th–75th percentile). Categorical variables are reported as frequencies and crude percentages (*n*, %). Ninety-five percent confidence intervals (95% CI) for crude proportions were calculated using the Clopper–Pearson exact binomial method, and confidence intervals for incidence rates per 100 person-years were derived assuming exact Poisson limits. Cumulative incidence curves for the competing terminal events of spontaneous passage and surgical reintervention were constructed using the Aalen–Johansen estimator. Within-patient paired stone burden progression between baseline 3-month NCCT and final cross-sectional NCCT was evaluated using the non-parametric Wilcoxon signed-rank test. All tests were two-tailed, and statistical significance was defined at *p* < 0.05. To account for unequal follow-up durations across subgroups and avoid exposure-time confounding, stone progression was also standardized as a growth rate, calculated as the absolute change in 2D stone burden divided by the total follow-up duration (reported as mm^2^/month and annualized to mm^2^/year).

## 3. Results

Time zero was defined as the 3-month postoperative NCCT scan that established study eligibility. Twenty-nine patients meeting the inclusion criteria, with a mean age of 50.10 ± 12.01 years, including 20 (69.0%) men and 9 (31.0%) women, were enrolled in this study. Comorbidities were present in 17 (58.6%) patients. Nineteen (65.5%) stones were in the left kidney, and the mean stone density was 1105.7 ± 116.1 HU. The median preoperative 2D stone burden was 332.0 mm^2^ (interquartile range [IQR]: 282.0–386.0 mm^2^)) and the median baseline RSF 2D burden measured at the 3-month postoperative evaluation was 24.0 mm^2^ (IQR: 13.0–33.0 mm^2^). The mean follow-up duration was 38.1 ± 18.8 months, and the median last pre-event/last follow-up 2D stone burden was 57.0 mm^2^ (IQR: 38.0–104.0 mm^2^). During the follow-up period, 15 patients (51.7%) required surgical intervention (four undergoing PCNL and eleven undergoing RIRS), whereas four (13.8%) experienced spontaneous stone passage and 10 (34.5%) remained on active surveillance. The patients’ sociodemographic and clinical characteristics, morphological stone data, and postoperative follow-up outcomes are shown in detail in Table 1.

**Figure 2:**
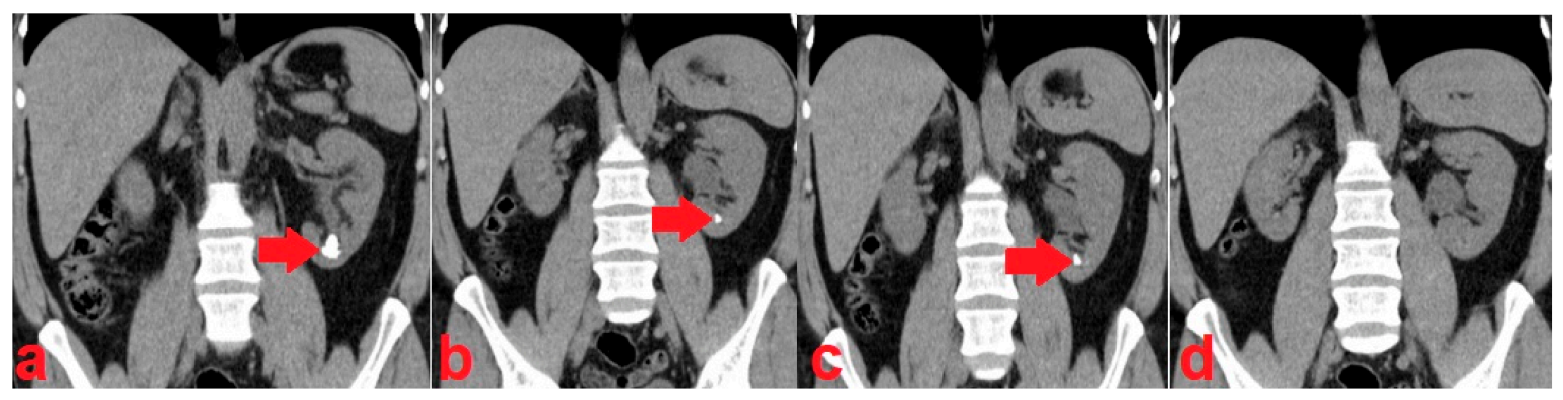
Serial non-contrast computed tomography (NCCT) images of a patient who achieved documented spontaneous fragment passage: (**a**) Preoperative NCCT demonstrating an isolated left lower-pole calculus measuring 17.0 mm in maximum diameter (arrow); (**b**) Baseline NCCT obtained at 3 months postoperatively (study time zero) confirming eligibility with a persistent 5.0 mm residual stone fragment (arrow); (**c**) Persistence of the calculus (arrow) on NCCT performed at 12 months postoperatively; and (**d**) Final NCCT at 18 months postoperatively confirming complete spontaneous stone clearance (terminal stone burden: 0.0 mm^2^).

Spontaneous resolution was observed in four patients (13.8%) during the follow-up period. The mean follow-up time in these cases was 16.5 ± 1.73 months. In patients achieving spontaneous fragment clearance, the median baseline 2D stone burden was 7.5 mm^2^ (IQR: 6.0–10.8 mm^2^) at the commencement of follow-up, reached 10.5 mm^2^ (IQR: 9.0–13.0 mm^2^) on the last positive evaluation prior to passage, and then ended at a terminal stone burden of 0.0 mm^2^ (IQR: 0.0–0.0 mm^2^) at final follow-up. Radiological images of a patient with spontaneous resolution are shown in Figure 2.

In the active surveillance group (*n* = 10), all patients (100%) exhibited radiological progression, demonstrating a >20% increase in 2D stone burden compared with baseline measurements over a mean follow-up of 28.8 ± 11.67 months. In this subgroup, the median 2D stone burden progressed from a baseline of 16.5 mm^2^ (IQR: 13.8–19.8 mm^2^) to a last follow-up burden of 46.5 mm^2^ (IQR: 34.2–50.0 mm^2^), representing a median absolute (Δ) increase of 25.5 mm^2^ (IQR: 22.5–32.5 mm^2^).

**Figure 3:**
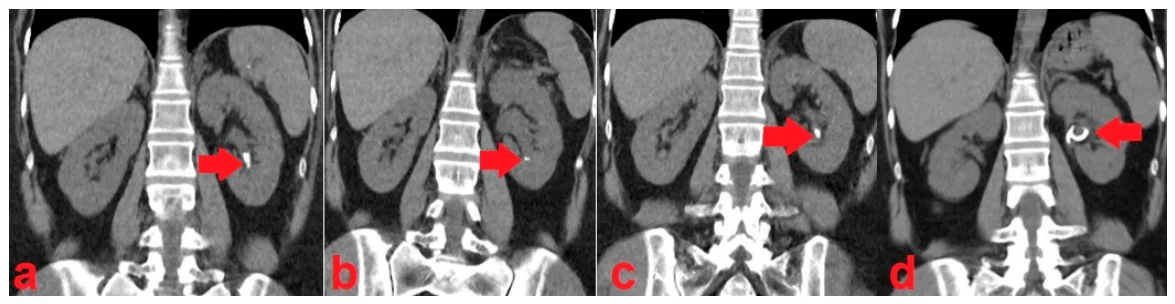
Serial non-contrast computed tomography (NCCT) images of a patient requiring secondary retrograde intrarenal surgery (RIRS): (**a**) Preoperative NCCT demonstrating an isolated lower-pole calculus (arrow); (**b**) Baseline NCCT obtained at 3 months postoperatively establishing eligibility with a 4.0 mm residual stone fragment; (**c**) Follow-up NCCT at 39 months postoperatively revealing interval stone enlargement to 9.0 mm (arrow); and (**d**) Control NCCT confirming complete stone clearance following secondary RIRS at 42 months postoperatively (arrow: JJ stent) (terminal stone burden: 0.0 mm^2^).

**Figure 4:**
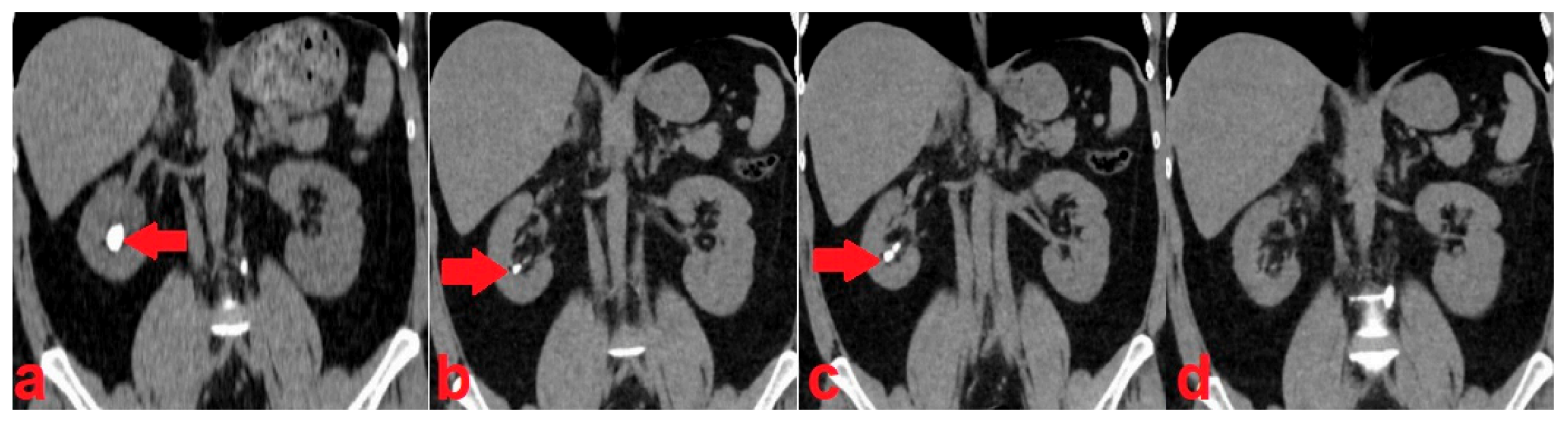
Serial radiological imaging of a patient managed with secondary percutaneous nephrolithotomy (repeat PCNL): (**a**) Preoperative non-contrast computed tomography (NCCT) demonstrating an isolated left lower-pole calculus measuring 20.0 mm in maximum diameter (arrow); (**b**) Baseline NCCT obtained at 3 months postoperatively (study time zero) showing a 6.0 mm residual stone fragment (arrow) establishing study eligibility; (**c**) Surveillance NCCT at 60 months postoperatively revealing interval fragment enlargement to 15.0 mm (arrow); and (**d**) Control NCCT confirming complete stone clearance following secondary PCNL at 63 months postoperatively (terminal stone burden: 0.0 mm^2^).

Secondary endourological interventions due to RSFs were performed on 15 (51.7%) patients. Four of these patients underwent PCNL, and 11 underwent RIRS. Two patients underwent RIRS under emergency conditions during follow-up, while surgery was performed under elective conditions on the remaining 13. Surgery was performed on eight of the elective surgery group due to stone progression during follow-up, and on five due to the development of stone-related symptoms such as persistent lateral pain or hematuria. The mean follow-up time in the cases undergoing endourological intervention was 50 ± 16.4 months. The median preoperative 2D stone burden was 33.0 mm^2^ (IQR: 25.0–40.0 mm^2^), increasing markedly to a pre-procedural burden of 104.0 mm^2^ (IQR: 65.0–147.5 mm^2^) prior to surgical treatment, ultimately achieving a terminal stone burden of 0.0 mm^2^ (IQR: 0.0–0.0 mm^2^) following successful re-intervention. Due to stone hardness and unsuitable calyceal anatomy, SWL therapy was not recommended or performed on any patient scheduled for secondary intervention. Radiological images of a patient who underwent RIRS as a result of the decision to perform surgery during follow-up are shown in Figure 3, and images of another patient who underwent PCNL are shown in Figure 4.

Over a total follow-up duration of 1104.0 person-months (92.0 person-years) with a median follow-up of 36 months (IQR: 18–48 months), the incidence rate of secondary surgical intervention was 16.3 per 100 person-years (95% confidence interval [CI]: 9.1–26.9). The incidence rate of spontaneous stone passage was 4.3 per 100 person-years (95% CI: 1.2–11.1). At the final follow-up evaluation, spontaneous stone passage occurred in 13.8% of patients (95% CI: 3.9–31.7%), whereas secondary surgical intervention was required in 51.7% (95% CI: 32.5–70.6%) due to symptom development or documented fragment progression. In the competing-risk cumulative incidence analysis (Aalen–Johansen estimator) treating spontaneous passage and surgical intervention as competing terminal events, the cumulative incidence of spontaneous clearance plateaued at 14.7% by 18 months. Conversely, the cumulative incidence of secondary surgical intervention progressively increased to 17.1% at 36 months, 39.1% at 45 months, 52.3% at 60 months, and 85.3% at 72 months (median time to reintervention: 60 months).

When standardized for variable exposure durations, the median annualized stone growth rate across the overall cohort was 11.7 mm^2^/year (IQR: 6.8–18.4 mm^2^/year). Annualized progression was slowest in patients achieving spontaneous passage (2.2 mm^2^/year, IQR: 1.6–2.4), intermediate in those on active surveillance (11.3 mm^2^/year, IQR: 8.2–14.6), and fastest in the secondary reintervention subgroup (17.6 mm^2^/year, IQR: 11.8–24.2).

The baseline 2D stone burden was median 24.0 mm^2^ (IQR: 13.0–33.0 mm^2^), which increased to median 57.0 mm^2^ (IQR: 38.0–104.0 mm^2^) on the last positive evaluation. Across the entire cohort, interval stone progression from baseline to the last scan was statistically significant (median absolute increase: 35.0 mm^2^, IQR: 22.0–66.0 mm^2^; paired Wilcoxon signed-rank test: W = 0.0, *p* < 0.001). For all patients achieving spontaneous passage or undergoing definitive secondary intervention, the terminal stone burden at final evaluation was confirmed radiologically as 0.0 mm^2^ (IQR: 0.0–0.0 mm^2^) (complete stone clearance).

Perioperative complications associated with the index-PCNL procedure (≤30 days postoperatively) were observed in four patients (13.8%, Clavien–Dindo ≥ Grade III). These included prolonged urinary extravasation requiring retrograde JJ stent insertion in two cases (6.9%, Grade IIIa), symptomatic retroperitoneal urinoma necessitating percutaneous drainage catheter placement in one case (3.4%, Grade IIIa), and postoperative acute pulmonary edema requiring temporary intensive care unit admission in one case (3.4%, Grade IVa). No perioperative mortality or Grade IVb complications occurred. During longitudinal surveillance, residual-fragment-related adverse clinical events developed in eight patients (27.6%). These events comprised: acute ureteral fragment migration with refractory renal colic requiring emergency semi-rigid ureterorenoscopy (URS) with fragment retrieval in one patient (3.4%, Grade IIIb); acute intractable renal colic and obstruction requiring emergency secondary RIRS in two patients (6.9%, Grade IIIb); and persistent or recurrent dull flank pain or gross/microscopic hematuria prompting elective secondary surgical intervention in five patients (17.2%). Regarding secondary intervention outcomes (*n* = 15), 11 patients (73.3%) underwent RIRS and four patients (26.7%) underwent repeat PCNL. Complete single-stage stone clearance (terminal stone burden: 0.0 mm2) was radiologically confirmed by cross-sectional NCCT in all 15 cases (100.0%), without the need for staged auxiliary procedures. No major perioperative complications (≥Grade III) occurred following secondary surgical procedures.

## 4. Discussion

Due to the unique anatomy of the lower calyces (a narrow infundulopelvic angle, and a long, narrow infundibulum), lower pole renal stones constitute the stone group with the lowest likelihood of spontaneous passage and the highest risk of residual fragments following endourological intervention [6,10]. EAU guidelines recommend PCNL surgery as the primary option in large lower pole kidney stones, with treatment success being determined not only by the stone-free rate during surgery, but also by the natural course of RSFs in the postoperative period [4,6]. While studies in the literature contain satisfactory information regarding the progression, symptom development, and secondary intervention requirements in RSFs ≤ 4 mm in size, data regarding those > 4 mm in size forming due to an isolated lower pole kidney stone are relatively limited. The present study evaluated the longitudinal outcomes of 29 patients who underwent PCNL for lower pole calculi and presented with RSFs > 4.0 mm (median baseline 2D stone burden: 24.0 mm^2^, IQR: 13.0–33.0 mm^2^) at the 3-month postoperative evaluation over a median follow-up duration of 36 months (IQR: 18–48 months). More than half of the patients (51.7%) required secondary endourological intervention, with pre-operative imaging documenting substantial stone enlargement in this surgical subgroup (median: 104.0 mm^2^, IQR: 65.0–147.5 mm^2^), whereas patients remaining on active surveillance exhibited a more moderate progression (median: 46.5 mm^2^, IQR: 34.2–50.0 mm^2^).

Large-series studies have reported that the natural course of residual fragments remaining following PCNL or RIRS varies depending on the stone and the location thereof. For example, in their multicenter study, Hein et al. reported that residual fragments remaining following endourological procedures resulted in stone-related clinical events (pain, infection, or growth) at rates between 20% and 43% at long-term follow-up [11]. In the present study, the median baseline 2D stone burden in the 10 patients (34.5%) managed with active surveillance was 16.5 mm^2^ (IQR: 13.8–19.8 mm^2^), which increased to 46.5 mm^2^ (IQR: 34.2–50.0 mm^2^) at the final follow-up evaluation, representing a median absolute increase (Δ) of 25.5 mm^2^ (IQR: 22.5–32.5 mm^2^). Consistent with our results, Brain et al. documented a 60.0% growth rate among residual stone fragments > 4.0 mm in maximum stone diameter, with lower pole localization significantly predisposing fragments to enlargement [12]. In a recent PCNL series, even for fragments measuring <4.0 mm in diameter, traditionally regarded as clinically insignificant, led to symptomatic events and intra-stone growth at levels as high as 58% [8]. The gravity-related anatomical disadvantage of the lower pole adversely impacts urine flow dynamics, allowing lithogenic crystals in urine to precipitate onto the existing fragment more easily, thus laying the foundation for rapid stone area growth. In addition, the perpendicular anatomy of the lower pole makes it difficult for fragments to enter directly into the pelvicalyceal system, and causes them to be retained in the pole. These conditions explain the basic physiological mechanism behind the RSF progression and low spontaneous passage rates.

One of the principal findings of this study was that 51.7% of patients (*n* = 15) required secondary endourological intervention (four PCNL, 11 RIRS) during follow-up. In this subgroup, the median follow-up duration was 45 months (IQR: 39–64.5 months), and the median 2D stone burden increased from a baseline of 33.0 mm^2^ (IQR: 25.0–40.0 mm^2^) to a pre-procedural burden of 104.0 mm^2^ (IQR: 65.0–147.5 mm^2^) prior to surgical retreatment. Reported re-intervention rates in the follow-up of RSFs in previous studies range from 16% to 19% in the first year, from 28% to 34% at the end of 2–3 year follow-up, and rise up to 73% during prolonged follow-up of 4.8 years [8,10,13,14,15]. This dynamic rising trend closely resembles the clinical outcomes in the present study.

The majority of our patients scheduled for secondary surgery (86.6%) were operated under elective conditions due to pronounced stone progression in the asymptomatic period, and RIRS was only performed under emergency conditions on two patients (13.4%). Studies with a similar content to the current research, but investigating RSFs in all kidney stones rather than solely in the lower calyx, have reported emergency re-intervention rates of 15.6–26.3% [10,16]. From that perspective, this study is compatible with the previous literature. Although elective reintervention was performed in the majority of surgical cases (86.7%) before acute obstructive complications or urosepsis developed, our single-arm observational design cannot definitively establish a causal protective effect of routine surveillance.

In our cohort, 51.7% of patients (*n* = 15) underwent secondary endourological intervention during follow-up under our center’s structured surveillance strategy. It is essential to recognize that this reintervention proportion reflects both intrinsic stone progression dynamics and institutional practice patterns within a shared decision-making framework. While EAU guidelines currently issue a weak recommendation for the active treatment of RSFs > 4.0 mm, our findings provide tangible evidence regarding the consequences of initial observation: 100.0% of surveillance cases exhibited interval growth, and over half ultimately required surgery. Nevertheless, our single-center experience should not be construed as a universal mandate for immediate re-treatment in every patient, but rather as supportive evidence that conservative management of lower pole RSFs > 4.0 mm necessitates rigorous, proactive monitoring due to high rates of delayed intervention.

In the present study, spontaneous passage occurred in 13.8% (*n* = 4, 95% CI: 3.9–31.7%) of patients with lower pole RSFs > 4.0 mm, reaching a cumulative incidence of 14.7% at 18.0 months. The observation that these patients presented with smaller baseline 2D stone burdens (median: 7.5 mm^2^, IQR: 6.0–10.8 mm^2^) is exploratory and hypothesis-generating, suggesting that fragments closer to the 4.0 mm threshold may have a higher propensity for spontaneous clearance. However, given the limited number of clearance events (*n* = 4), this trend warrants cautious interpretation and validation in larger prospective cohorts. Previous long-term series evaluating post-PCNL residual fragments across all renal locations have reported higher spontaneous passage proportions of 21–23% [16,17]. The comparatively lower clearance observed in our cohort is likely attributable to the strict inclusion of isolated lower pole calculi exceeding 4.0 mm in diameter, where dependent calyceal spatial anatomy further impedes natural expulsion.

Eleven (73.3%) of the 15 patients subjected to secondary interventions underwent RIRS, while secondary PCNL was applied to four (26.7%). Recent advances in RIRS technology, including the increased effectiveness of flexible ureterorenoscopes and high-power laser lithotriptors, have led to RIRS assuming the prominent role in the secondary treatment of RSFs, and this has been shown to be capable of safe application with high success and low morbidity rates [18]. Similarly in the present study, RIRS was employed in the great majority of cases scheduled for secondary intervention under elective conditions, while secondary PCNL was employed in the limited number of cases with a large stone burden or inaccessible to RIRS. SWL was not considered or applied as a treatment option due to high stone hardness (average 1105.7 HU) or negative calyx anatomy in any case. This is entirely consistent with the low efficacy of SWL, as also stated in the current guidelines, in high-density stones located in the lower pole [4].

Although this study provides valuable insights into the longitudinal trajectory of lower pole RSFs > 4.0 mm post-PCNL, several limitations warrant consideration. First, its retrospective, single-center design and modest cohort size (N = 29) inherently limit statistical power, widen confidence intervals, and restrict broad generalizability. In a sample of this size, individual clinical events exert a noticeable influence on crude percentages; therefore, the reported incidence rates and event proportions must be interpreted cautiously as descriptive and exploratory rather than definitive epidemiological estimates. Of the 33 initial patients identified with RSFs at 3 months, four were excluded due to unrelated mortality (*n* = 1), incomplete baseline documentation (*n* = 1), or loss to follow-up (*n* = 2), which introduces potential attrition and selection bias. Furthermore, our findings are subject to inherent indication bias (confounding by indication). Because elective secondary reinterventions were clinically triggered by documented interval enlargement (>20% increase) or the onset of stone-related symptoms, the higher terminal burdens and annualized growth rates observed in the reintervention subgroup partly reflect clinical selection criteria rather than an unconstrained biological natural history. Second, because the recruitment window spanned approximately a decade, subtle temporal evolutions in endourological instrumentation, surgical experience, and clinical decision-making may have occurred. Third, specific lower-pole anatomical metrics (infundibulopelvic angle, infundibular length, and infundibular width) were not quantitatively evaluated, precluding direct correlations between calyceal spatial geometry and clearance kinetics. Finally, comprehensive 24-h urinary metabolic profiles, dietary records, and crystallographic stone composition analyses were unavailable, preventing the evaluation of biochemical determinants or targeted pharmacological metaphylaxis on fragment behavior. Conversely, a major methodological strength of this study is the mandatory utilization of cross-sectional NCCT at both the 3-month baseline and the final clinical endpoint across all 29 patients (100.0%). By definitively establishing all terminal outcomes—including complete spontaneous clearance, pre-operative reintervention planning, and final active surveillance status—with NCCT, we eliminated inter-modality measurement bias in paired stone burden analyses. Additional strengths include the evaluation of a highly homogeneous cohort restricted exclusively to isolated lower pole calculi and a substantial median follow-up of 36.0 months (IQR: 18.0–48.0 months).

## 5. Conclusions

In this selected single-center cohort of 29 patients with lower-pole residual fragments > 4.0 mm at 3-month NCCT after PCNL, 15 patients underwent secondary reintervention and four achieved documented spontaneous stone passage during variable longitudinal follow-up. Given the modest sample size, wide confidence intervals, and individualized monitoring intervals, these descriptive findings should be interpreted cautiously and do not establish definitive thresholds or the optimal timing for treatment. Adequately powered, prospective multicenter cohorts with standardized cross-sectional imaging protocols are required to validate these long-term progression dynamics.

## Figures and Tables

**Table 1:** Patients’ demographic features, stone characterization, and perioperative and postoperative outcomes.

Variables	Overall (N = 29)	Spontaneous Passage (*n* = 4)	Active Surveillance (*n* = 10)	Secondary Reintervention (*n* = 15)
Age (years) *	50.1 ± 12.0	54.8 ± 5.7	45.4 ± 11.9	52.0 ± 12.8
BMI (kg/m^2^) *	28.4 ± 4.0	27.4 ± 5.0	28.6 ± 5.3	28.5 ± 3.0
Sex, *n* (%)				
Female	9 (31%)	2 (50%)	3 (30%)	4 (26.7%)
Male	20 (69%)	2 (50%)	7 (70%)	11 (73.3%)
Comorbidities, *n* (%)	17 (58.6%)	2 (50%)	5 (50%)	10 (66.7%)
Diabetes mellitus	5 (17.2%)	-	1 (10%)	4 (26.7%)
Hypertension	8 (27.5%)	2 (50%)	3 (30%)	3 (20%)
Coronary disease	2 (6.9%)	-	-	2 (13.3%)
Respiratory disorder	2 (6.9%)	-	1 (10%)	1 (6.7%)
Stone laterality, *n* (%)				
Right	10 (34.5%)	2 (50%)	3 (30%)	5 (33.3%)
Left	19 (65.5%)	2 (50%)	7 (70%)	10 (66.7%)
Preoperative 2D stone burden (mm^2^) ^†^	332.0 (282.0–386.0)	381.5 (345.5–394.5)	330.0 (288.0–365.2)	332.0 (277.0–415.5)
ASA score				
ASA I (*n*, %)	6 (20.7%)	1 (25%)	3 (30%)	2 (13.3%)
ASA II (*n*, %)	21 (72.4%)	3 (75%)	5 (50%)	13 (86.7%)
ASA III (*n*, %)	2 (6.9%)	-	2 (20%)	-
Radiopacity, *n* (%)				
Radio-opaque	26 (89.7%)	4 (100%)	10 (100%)	12 (80%)
Radiolucent	3 (10.3%)	0 (0.0%)	0 (0.0%)	3 (20%)
Stone density (HU) *	1105.7 ± 116.1	1102.7 ± 94.2	1170.7 ± 135.5	1063.2 ± 90.9
Operative time (min) *	83.8 ± 30.3	111.3 ± 41.1	83.0 ± 37.9	77.0 ± 16.6
Hospital stay (days) *	3.4 ± 2.3	3.0 ± 0.8	3.7 ± 3.7	3.3 ± 1.0
Nephrostomy duration (days) *	3.0 ± 1.7	2.8 ± 0.5	3.3 ± 2.8	2.9 ± 0.6
Baseline 3-month 2D stone burden (mm^2^) ^†^	24.0 (13.0–33.0)	7.5 (6.0–10.8)	16.5 (13.8–19.8)	33.0 (25.0–40.0)
Last pre-event/last follow-up 2D stone burden (mm^2^) ^†^	57.0 (38.0–104.0)	10.5 (9.0–13.0)	46.5 (34.2–50.0)	104.0 (65.0–147.5)
Absolute change 2D stone burden (mm^2^) ^†^	35.0 (22.0–66.0)	3.0 (2.2–3.0)	25.5 (22.5–32.5)	66.0 (40.5–106.0)
Annualized stone growth rate (mm^2^/year)	11.7 (6.8–18.4)	2.2 (1.6–2.4)	11.3 (8.2–14.6)	17.6 (11.8–24.2)
Terminal 2D stone burden at final evaluation (mm^2^) ^†^	0.0 (0.0–38.0)	0.0 (0.0–0.0)	46.5 (34.2–50.0)	0.0 (0.0–0.0)
Follow-up duration (months) ^†^	36 (18–48)	16.5 (15–18)	27 (18–36)	45 (39–64.5)

* Data are presented as mean ± standard deviation. ^†^ Data are presented as median and interquartile range (IQR, 25th–75th percentile). Percentages (*n*, %) represent column-based proportions calculated within each subgroup column. In this cohort, comorbidity subcategories were mutually exclusive (each patient presented with a single primary condition); hence, subcategory counts sum directly to the total comorbidity count (*n* = 17, 58.6%). Abbreviations: 2D, two dimensional; ASA, American Society of Anesthesiologists; BMI, body mass index; HU, Hounsfield unit; N/*n*, number of patients; PCNL, percutaneous nephrolithotomy.

## Data Availability

The data presented in this study are available on request from the corresponding author. The data are not publicly available due to patient privacy protection.

## Supplementary Materials

The following supporting information can be downloaded at https://www.mdpi.com/article/10.3390/jcm15187060/s1, Table S1. Patient-level demographic characteristics, baseline stone parameters, longitudinal radiological measurements, clinical events, and final outcomes.

## Abbreviations

The following abbreviations are used in this manuscript:

RSF	Residual stone fragments
PCNL	Percutaneous nephrolithotomy
EAU	European Association of Urology
SWL	Shock wave lithotripsy
RIRS	Retrograde intrarenal surgery
CT	Computed tomography
ASA	American Society of Anesthesiologists
HU	Hounsfield units
KUB	Kidney, ureter, and bladder
