# Supplementary material for: Natural History and Reintervention After PCNL for Isolated Lower-Pole Stones with Residual Fragments > 4 mm: A Retrospective Cohort Study
Source: J Clin Med. 2026 Sep 11;15(18):7060. doi: 10.3390/jcm15187060 (PMC13607568; doi:10.3390/jcm15187060)
Supplement: Supplementary file 1 [file jcm-15-07060-s001.zip › jcm-4531329-supplementary.pdf]

Table S1. Patient-level demographic characteristics, baseline stone parameters, longitudinal radiological measurements, clinical events, and final outcomes

Patient number	Sex	Age	ASA	Laterality	Density (HU)	Preoperative 2D stone burden (mm^2)	Operative time (minute)	Clinical trajectory / event time (month)	Baseline 3-month 2D stone burden (mm^2)	Clinical trajectory / event	Last pre-event / final 2D stone burden (mm^2)	Absolute change (mm^2)	Terminal stone burden at final evaluation (mm^2)	Last imaging modality
1	F	47	1	Right	1025	420	125	15	6	SP	9	3	0	NCCT
2	M	55	2	Left	1142	330	82	36	16	AS	38	22	22	NCCT
3	F	50	2	Left	1245	306	45	15	9	AS	33	24	24	NCCT
4	M	63	2	Left	940	451	70	36	13	RIRS	56	43	0	NCCT
5	F	58	2	Left	1038	377	135	18	6	SP	9	3	0	NCCT
6	M	53	2	Left	1068	471	65	42	24	RIRS	57	33	0	NCCT
7	M	30	1	Left	980	372	55	36	19	AS	50	31	31	NCCT
8	M	46	2	Left	1056	236	70	63	24	PCNL	177	153	0	NCCT
9	M	55	2	Right	1280	345	80	15	16	AS	49	33	33	NCCT
10	M	33	2	Right	1145	314	55	42	13	RIRS	57	44	0	NCCT
11	M	51	3	Left	965	177	68	15	9	AS	33	24	24	NCCT
12	M	60	2	Right	1230	251	135	18	9	SP	12	3	0	NCCT
13	F	63	2	Right	1185	328	110	60	26	PCNL	173	147	0	NCCT
14	M	70	2	Left	1088	330	80	36	33	RIRS	71	38	0	NCCT
15	M	47	1	Right	1155	345	80	45	38	RIRS	104	66	0	NCCT
16	F	41	2	Left	998	471	90	60	44	RIRS	122	78	0	NCCT
17	M	54	2	Right	1118	386	50	15	16	SP	16	0	0	NCCT
18	M	59	3	Left	1210	282	100	27	13	AS	22	9	9	NCCT
19	M	53	2	Left	1074	380	60	33	27	RIRS	79	52	0	NCCT
20	F	60	1	Left	982	230	75	45	38	RIRS	110	72	0	NCCT
21	M	67	2	Right	1060	332	65	66	66	PCNL	200	134	0	NCCT
22	F	29	1	Left	1180	397	85	42	24	AS	63	39	39	NCCT
23	M	28	1	Left	1281	264	55	27	20	AS	55	35	35	NCCT
24	M	52	2	Right	1052	330	80	48	17	AS	44	27	27	NCCT
25	M	46	2	Left	1245	240	85	15	42	RIRS	64	22	0	NCCT
26	M	26	2	Right	1028	336	60	72	80	PCNL	239	159	0	NCCT
27	M	47	2	Left	1010	468	110	66	28	RIRS	66	38	0	NCCT
28	F	65	2	Left	915	236	80	69	33	RIRS	104	71	0	NCCT
29	F	45	2	Right	1372	452	180	27	33	AS	50	17	17	NCCT

Abbrevations: 2D, two dimensional; ASA, American Society of Anesthesiologists; BMI, body mass index; HU, Hounsfield unit.
